# Restoration of WNT4 inhibits cell growth in leukemia-derived cell lines

**DOI:** 10.1186/1471-2407-13-557

**Published:** 2013-11-25

**Authors:** Beatriz García-Castro, Monserrat Alvarez-Zavala, Alma R Riveros-Magaña, Pablo C Ortíz-Lazareno, Sarah Ratkovich-González, Georgina Hernández-Flores, Alejandro Bravo-Cuellar, Luis F Jave-Suarez, Adriana Aguilar-Lemarroy

**Affiliations:** 1 División de Inmunología, Centro de Investigación Biomédica de Occidente (CIBO), Instituto Mexicano del Seguro Social (IMSS), Sierra Mojada No. 800, Col. Independencia, 44340 Guadalajara, Jalisco, Mexico; 2 Programa de Doctorado en Ciencias Biomédicas, Centro Universitario de Ciencias de la Salud (CUCS) - Universidad de Guadalajara, Guadalajara, Jalisco, Mexico; 3 Programa de Doctorado en Investigación Clínica, CUCS - Universidad de Guadalajara, Guadalajara, Jalisco, Mexico

**Keywords:** WNT4, WNT signaling, Leukemia, Hematopoietic malignancies, Non-canonical pathway, FZD6

## Abstract

**Background:**

WNT signaling pathways are significantly altered during cancer development. Vertebrates possess two classes of WNT signaling pathways: the “canonical” WNT/β-catenin signaling pathway, and the “non-canonical” pathways including WNT/Ca2^+^ and WNT/Planar cell polarity [PCP] signaling. WNT4 influences hematopoietic progenitor cell expansion and survival; however, WNT4 function in cancer development and the resulting implications for oncogenesis are poorly understood.

The aim of this study was twofold: first, to determine the expression of WNT4 in mature peripheral blood cells and diverse leukemia-derived cells including cell lines from hematopoietic neoplasms and cells from patients with leukemia; second, to identify the effect of this ligand on the proliferation and apoptosis of the blast-derived cell lines BJAB, Jurkat, CEM, K562, and HL60.

**Methods:**

We determined WNT4 expression by quantitative reverse transcriptase-polymerase chain reaction (qRT-PCR) in peripheral blood mononuclear cells (PBMCs) and T- and B-lymphocytes from healthy individuals, as well as from five leukemia-derived cell lines and blasts derived from patients with leukemia. To analyze the effect of WNT4 on cell proliferation, PBMCs and cell lines were exposed to a commercially available WNT4 recombinant human protein. Furthermore, WNT4 expression was restored in BJAB cells using an inducible lentiviral expression system. Cell viability and proliferation were measured by the addition of WST-1 to cell cultures and counting cells; in addition, the progression of the cell cycle and the amount of apoptosis were analyzed in the absence or presence of WNT4. Finally, the expression of WNT-pathway target genes was measured by qRT-PCR.

**Results:**

WNT4 expression was severely reduced in leukemia-derived cell lines and blasts derived from patients with leukemia. The exposure of cell lines to WNT4 recombinant protein significantly inhibited cell proliferation; inducing WNT4 expression in BJAB cells corroborated this observation. Interestingly, restoration of WNT4 expression in BJAB cells increased the accumulation of cells in G1 phase, and did not induce activation of canonical WNT/β-catenin target genes.

**Conclusions:**

Our findings suggest that the WNT4 ligand plays a role in regulating the cell growth of leukemia-derived cells by arresting cells in the G1 cell cycle phase in an FZD6-independent manner, possibly through antagonizing the canonical WNT/β-catenin signaling pathway.

## Background

WNT pathways direct the specific activation of sets of genes regulating a plethora of cellular responses such as cell growth, differentiation, movement, migration, polarity, cell survival, and immune response. Chronic activation of gene transcription resulting from aberrant activation of WNT pathways has pathological consequences, and can contribute to tumorigenesis [1-3].

Signaling is initiated when WNT ligands bind to Frizzled (FZD) family receptors, activating a canonical signaling pathway in which the central player is a cytoplasmic protein called β-catenin. WNT ligands also activate the so-called “non-canonical” pathways that are β-catenin-independent. These non-canonical pathways include the planar cell polarity (PCP) pathway that stimulates cytoskeletal reorganization, and the WNT-Ca2^+^ pathway that leads to calcium mobilization [4]. In addition to FZD, several other receptors and co-receptors have been implicated in triggering WNT signaling, including LDL-related protein (LRP), receptor tyrosine kinase-like orphan receptor (ROR), receptor-like tyrosine kinase (RYK), and protein tyrosine kinase 7 (PTK7) [2,5,6]. WNT signaling has been implicated in the regulation of oncogenic growth in leukemias of both myeloid and lymphoid lineages [7,8]. The canonical WNT/β-catenin signaling pathway has shown to exert a positive effect on the control of hematopoietic cell proliferation, survival, and differentiation. However, abnormal activation of the WNT/β-catenin signaling pathway has been linked to the pathogenesis of many carcinomas and hematological malignancies [9-12].

Non-canonical pathways are more often associated with negative control of the proliferation and induction of cell differentiation [13]. In this sense, antagonism between the canonical and the non-canonical pathways may be an essential mechanism for preventing dysregulated self-renewal and oncogenesis.

WNT4 is a ligand that plays an important role in survival and hematopoietic progenitor cell expansion through non-canonical signals [14,15]. In addition, non-canonical WNT4 signals may be critical in preventing tumor initiation by controlling EAF1 and EAF2/U19 expression in zebrafish and mammals [16]. In contrast, WNT4 canonical signals aid in maintaining cell growth and survival in kidney epithelial cells [17]. Several reports have been published describing the low expression of WNT4 in cells derived from solid tumor compared with normal cells [18-20].

Variations in *WNT* gene expression and the related signaling molecules have been reported in hematological cancers [21-23]. However, the function of WNT4 in leukemia, to our knowledge, has not yet been described; therefore, the goal of our research was to determine the expression of the WNT4 ligand in leukemia-derived cells, the effect of its expression on cell growth and apoptosis, and the WNT signaling pathway activated in our cell model.

## Results

### WNT4 is poorly expressed in leukemia-derived cells

**Figure 1:**
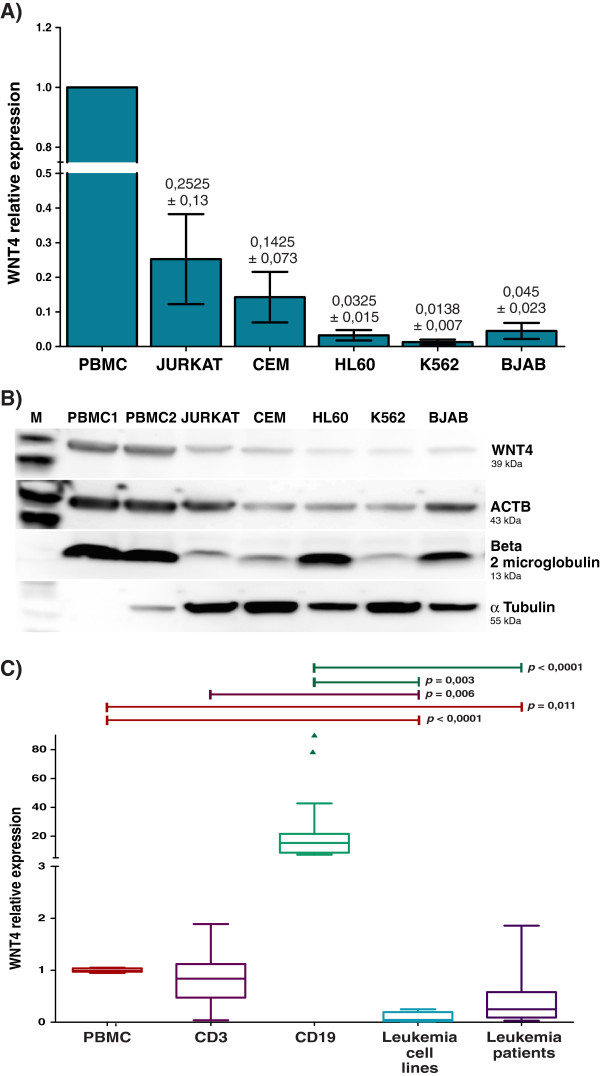
***WNT4 *expression in healthy and leukemia-derived cells. A)** Relative expression levels of *WNT4* were measured by qRT-PCR in PBMCs obtained from healthy volunteers (PBMC) and leukemia-derived cells lines (Jurkat, CEM, HL60, K562, and BJAB). An expression value of the PBMCs of one individual was set as 1. Analysis was calculated using ribosomal Protein L32 (*RPL32*), ribosomal Protein S18 (*RPS18*), and beta actin (*ACTB*) as reference genes. The graphic depicts the means and subsequent standard deviations (SD) obtained with all reference genes. **B)** Western blot assay showing the presence of WNT4 protein in PBMCs from two healthy individuals and leukemia-derived cells lines. ACTB, beta 2 microglobulin, and α tubulin were used as protein loading controls. **C)** Box plot graphic showing relative expression levels of *WNT4* measured by qRT-PCR normalized to the previously mentioned reference genes. The graph displays median (dark lines), 25–75th percentile (boxes), interquartile ranges (whiskers), and outliers (small, dark circles) from the CD3+ and CD19+ sorted cells of five healthy individuals, cell lines (Jurkat, CEM, HL60, K562, and BJAB), and 11 patients with leukemia. Average values from the PBMCs obtained from the five healthy volunteers were used as controls. Statistical significances are shown between both groups. Experiments were carried out at least twice.

Because WNT4 expression has been related with the hematopoietic cell proliferation and differentiation, we wanted to know whether abnormal immature leukemic cells express *WNT4*. To do this, we analyzed *WNT4* expression in BJAB, Jurkat, CEM, K562, and HL60 leukemia-derived cells. We compared the *WNT4* expression in these cells with the usual level of expression found in peripheral blood mononuclear cells (PBMCs) from healthy volunteers. We obtained complementary DNA (cDNA) from the leukemia-derived cells and the healthy PBMCs, and determined expression of *WNT4* by quantitative Reverse transcriptase-Polymerase chain reaction (qRT-PCR) in all samples. We used beta actin (*ACTB*), ribosomal protein L32 (*RPL32*), and ribosomal protein S18 (*RPS18*) as reference genes to normalize all values. Relative expression analysis was performed, setting the expression values from the PBMCs of one of the healthy volunteers as 1 (C1). As can be observed in Figure 1A, Jurkat and CEM cells, both of lymphoid origin, exhibit low expression of *WNT4* relative to C1 and C2, with relative values of 0.252 and 0.142, respectively. The lymphoblast-B BJAB cell line and myeloid types K562 and HL60 had the lowest expression, exhibiting nearly undetectable levels of WNT4 (0.045, 0.013 and 0.032, respectively) when compared with that of the controls.

To corroborate our observations, we analyzed WNT4 protein levels by western blot analysis in the leukemia-derived cell lines, and included protein obtained from two healthy individuals (PBMC1 and PBMC2) as controls (Figure 1B). We were able to detect a specific band of approximately 39KD that corresponded with the predicted weight for WNT4, mainly observed in the PBMCs; the WNT4 band was very weak in Jurkat, CEM, K562, and HL60 cell lines. We also probed for ACTB, beta 2 microglobulin, and α tubulin in the same blot to control for protein loading. Taken together, these results show that WNT4 expression in leukemia-derived cell lines is significantly decreased when compared with that of mature immune-system cells from clinically healthy individuals.

### WNT4 expression in T- and B-cells from healthy individuals and bone marrow cells from patients with leukemia

After demonstrating that *WNT4* expression is strongly reduced in leukemia-derived cell lines, we wanted to determine whether *WNT4* expression is also reduced in the bone marrow (BM) samples of patients with leukemia. Due to the origin of leukemia cell lines included in this study, we analyzed blasts from bone marrow of patients with acute lymphoblastic leukemia (ALL) and acute myeloblastic leukemia (AML). Additionally, to assess the contribution of lymphocytes to the *WNT4* expression observed in the PBMCs, we isolated T- and B-lymphocytes from five healthy individuals by flow cytometry sorting, and measured *WNT4* expression in these cells by real time-PCR. Normalization was performed using *ACTB*, *RPL32*, and *RPS18* as reference genes, and relative expression analysis of the ALL and AML bone marrow samples was performed using PBMCs as the control (set as 1).

Figure 1C shows that CD19+ cells are the major *WNT4* producing cells (~15–20-fold), and that CD3+ cells express levels similar to PBMCs (~0.86-fold). Interestingly, of the 11 BM cells from the patients with leukemia included in the study, ten showed very low expression of *WNT4*; this trend became more strongly evident when compared with the control CD19+ cells.

In summary, leukemia-derived cell lines and bone marrow cells from patients with leukemia show a statistically significant decrease in the expression of *WNT4* when compared with the expression in PBMCs from healthy individuals.

### Recombinant human WNT4 inhibits cell viability in leukemia

Because we showed that *WNT4* was more highly expressed in mature lymphocytes derived from healthy volunteers, and that its expression decreased in immature leukemia-derived cells, it was in our interest to determine the biological effects of WNT4 in leukemia-derived cells.

**Figure 2:**
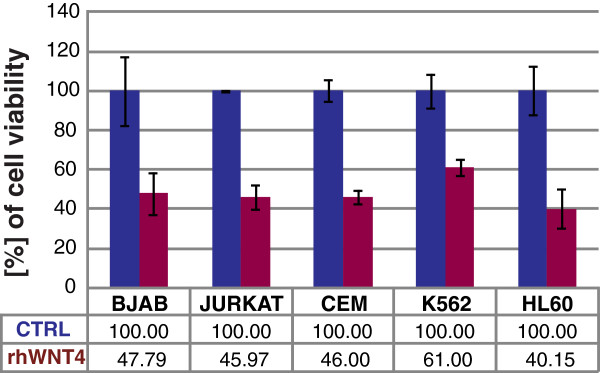
**rhWNT4 inhibits cell viability in leukemia-derived cell lines.** Percentage of cell viability was measured in leukemia-derived cell lines after stimulation with 200 ng/mL of recombinant human WNT4 (rhWNT4) for 24 h in culture. Percentage of cell viability were obtained using the optical density (OD) values of each unstimulated cell line as 100% cell proliferation. Percentage of cell proliferation was measured using WST-1 at 440 nm.

To do this we used BJAB, Jurkat, CEM, K562, and HL60 cell lines as models. We cultured 5 × 10^3^ cells in 96-well plates in the presence of 200 ng/mL of commercially available recombinant human WNT4 (rhWNT4). After 24 h of incubation, we measured the percentage of cell viability by adding WST-1 to the culture medium. Percentage of cell viability was calculated by taking the optical density (OD) values of cells, using the value from those without treatment as 100%. Incubation of leukemia-derived cells with rhWNT4 drastically affects their growth in culture; all tested cell lines exhibited a decrease in their percentage of cell viability when treated with rhWNT4 ranging from 61.00–40.15% (Figure 2).

### Restoration of WNT4 expression in BJAB cells inhibits cell growth

**Figure 3:**
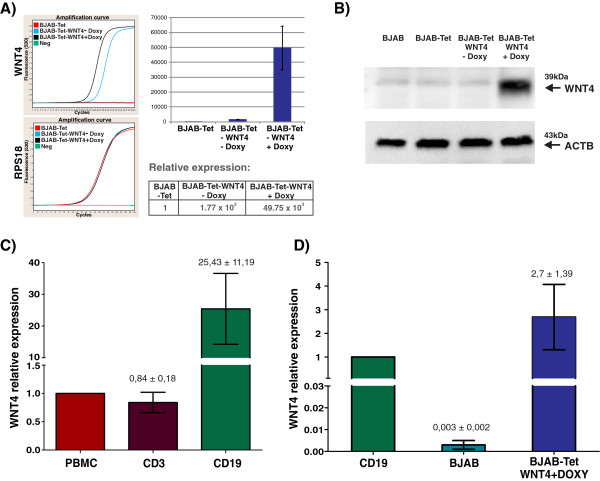
**Endogenous restoration of WNT4 in BJAB cells.** Overexpression of *WNT4* in the leukemia-derived BJAB cell line was performed by using a Doxycycline (Doxy)-inducible lentiviral expression system. After a 48-h incubation with Doxy, *WNT4* expression was confirmed using qRT-PCR **(A)**, and western blot assays **(B)**. **A)** Graphs showing the amplification curves obtained for *WNT4* and *RPS18* in BJAB-Tet-WNT4 cells cultured in the presence or absence of Doxy. The WNT4 normalized relative ratio was calculated utilizing the non-treated BJAB-Tet cells as a control (set as 1), and by using *ACTB*, *RPL32* and *RPS18* as reference genes in two independent experiments. **B)** Western blot assays (50 μg total protein) showing the presence of WNT4 protein after a 48-h incubation with Doxy; beta actin (ACTB) was used a protein loading control. **C** &**D)** Relative expression levels of *WNT4* were measured by qRT-PCR in PBMCs from healthy volunteers, CD3+ and CD19+ sorted cells obtained from healthy volunteers **(C)**, as well as in parental BJAB cells and in BJAB WNT4-expressing cells **(D)**. The average values of PBMCs (in **C**) and CD19+ sorted cells (in **D**) were used as controls. The graph depicts the means obtained with all reference genes ± standard deviations (SD).

To corroborate the effect observed on cell viability by the addition of rhWNT4 to leukemia-derived cell lines, we decided to restore *WNT4* expression in leukemia-derived cells. Because *WNT4* is expressed mainly in mature B cells, we thought that the best candidates to analyze the effect of *WNT4* restoration would be the BJAB cells, the only leukemia model cell line in our experiments that were CD19+. To restore *WNT4* expression, we used an inducible lentiviral expression system (Clontech, additional detail provided in Materials and Methods) in which the presence of Doxycycline (Doxy) activates the expression of *WNT4*. First, to determine whether *WNT4* was successfully expressed after Doxy treatment, RNA and total protein were obtained after 48-h of Doxy treatment; qRT-PCR and western blot assays were performed on these samples (see Figure 3). Relative *WNT4* expression was measured in BJAB-Tet cells infected with pLVX-Tet On vector alone, and in those infected with both pLVX-Tet On and pLVX-Tight-Puro-WNT4 vectors after cultivation in the presence or absence of Doxy for 48 h. Unexpectedly, as can be observed in Figure 3A, an increase in *WNT4* expression at the mRNA level was observed in the absence of Doxy (1.77 × 10^3^); however, *WNT4* expression increased strongly (49.75 × 10^3^-fold) in BJAB-Tet-WNT4 in the presence of Doxy. The *WNT4* normalized relative ratio was calculated using the values from BJAB-Tet cells as a control and *RPS18*, *RPL32*, and *ACTB* as reference genes. Furthermore, as shown in Figure 3B, WNT4 expression was confirmed at the protein level after 48 h of Doxy incubation. Despite the *WNT4* expression observed at the mRNA level in the BJAB-Tet-WNT4 cells cultivated in the absence of Doxy, WNT4 expression at the protein level could only be clearly observed after 48 h of Doxy treatment. To compare the expression levels obtained with our inducible *WNT4* system with those of normal cells, we performed qPCR on samples from PBMCs, T-cells (CD3+), B-cells (CD19+), BJAB-TET-WNT4 cells treated 48 h with Doxy, and parental BJAB cells. As can be observed in Figure 3C (left panel) and according to Figure 1C, relative *WNT4* expression (as normalized to the level in the average of PBMCs from five healthy individuals) showed a lower level of expression in CD3+ cells (0.84 ± 0.18-fold), while CD19+ cells express approximately 25.43 ± 11.19-fold higher. Furthermore, by normalizing to CD19+ cells (Figure 3C, right panel), we observed that BJAB-Tet-WNT4 cells in the presence of Doxy have a 2.7 ± 1.39-fold increase in *WNT4* expression. These results show that induction of *WNT4* in BJAB-Tet-WNT4 cells caused only ~2.5 times more expression of *WNT4* than the levels found in the B-cells of healthy subjects.

**Figure 4:**
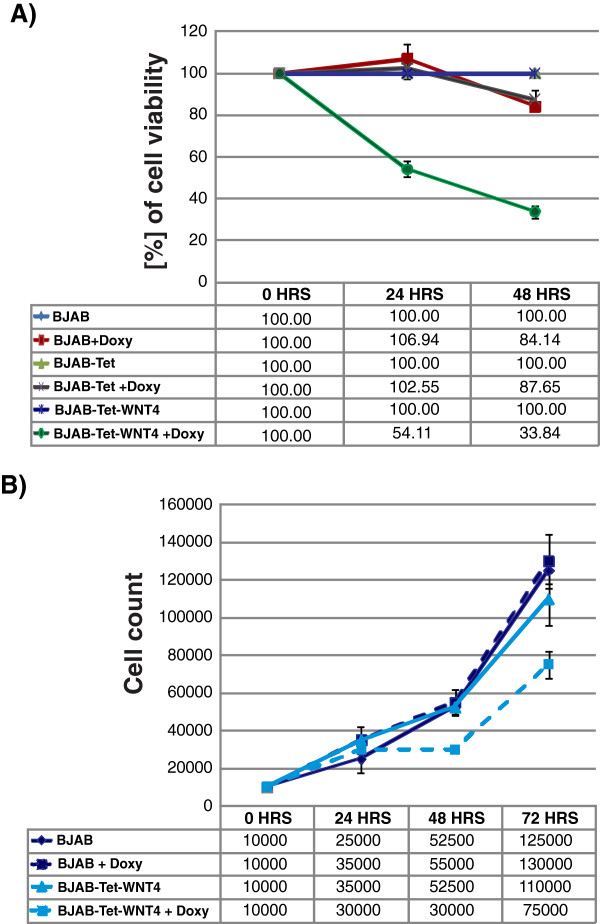
**Restoration of WNT4 in BJAB inhibits cell growth. A)** Percentage of cell viability was measured in BJAB-Tet-WNT4 cells after 24- and 48-h incubations with Doxycycline (Doxy). We also included parental BJAB and BJAB-Tet cells as controls. Percentage of cell viability was measured by adding WST-1 to the cell cultures for 2 h and reading the absorbance of treated and untreated cells at 440 nm. The value of each cell line of non-treated cells was set as 100% of cell proliferation. **B)** BJAB-Tet-WNT4 cells cultivated for 72 h in the presence or absence of Doxy were counted every 24 h. The parental BJAB cell line was included as control.

After ensuring that *WNT4* was successfully expressed in our inducible model, we examined the effect of this ligand on BJAB-cell viability and proliferation. We cultured parental BJAB, BJAB-Tet, and BJAB-Tet-WNT4 cells in the absence or presence of Doxy for 24- and 48 h. Subsequently, WST-1 was added to cell cultures and incubated for 2 h at 37°C before absorbance was read at 440 nm. Percentage of cell viability was calculated using the OD values of cells growing in the absence of Doxy as 100% (0 h). As shown in Figure 4, Doxy induces a very weak increase in cell viability at 24 h and reduces the viability rate slightly after 48 h in parental BJAB and BJAB-Tet cells. However, a strongly evident decrease in cell viability was observed in BJAB-Tet-WNT4 cells after 24-h (54.11%) and 48-h (33.84%) incubation with Doxy. The latter result suggests that WNT4 expression in BJAB cells reduces their viability; however, because WST-1 assays indicate the number of metabolically active cells, we asked whether cell proliferation was also affected by WNT4 expression. To do this, parental BJAB and BJAB-Tet-WNT4 cells were cultured in the absence or presence of Doxy for 72 h, and the number of cells was measured for each 24-h period. As can be observed in the cell counts plotted in Figure 4B, the growth of parental BJAB cells with or without Doxy exhibit similar proliferation curves, indicating that Doxy did not modify the growth of parental BJAB cells. The growth of BJAB-Tet-WNT4 cells in the absence of Doxy exhibits behavior similar to that observed in parental BJAB cells; in contrast, cell growth in BJAB-Tet-WNT4 cells was drastically decreased after 24 h of incubation with Doxy (see cell counts at 24 and 48 h). Note that the first 24-h of BJAB-Tet-WNT4 cell growth with or without Doxy follows nearly the same proliferation rate; one reason for this observation is that WNT4 ligand production and secretion takes at least 24 h after the addition of Doxy. Furthermore, we observed that after 48 h, Doxy-treated BJAB-Tet-WNT4 cells recovered proliferation, but at a lower rate. Overall, these results confirm the anti-proliferative role played by WNT4 in BJAB cells.

### WNT4 does not promote apoptosis, but strongly modulates the cell cycle

**Figure 5:**
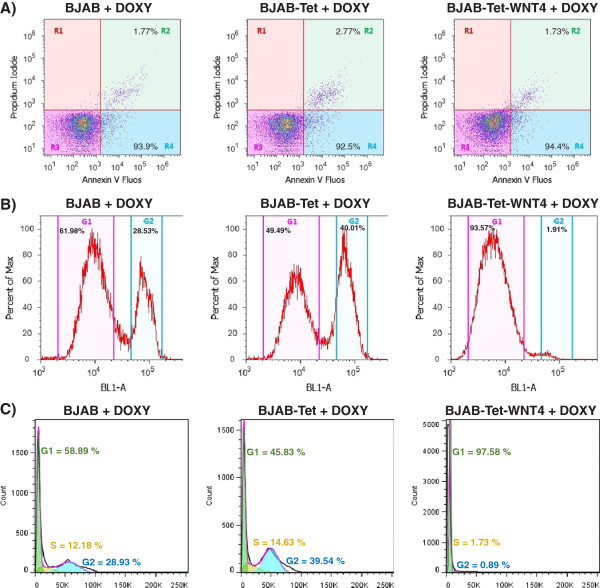
**Restoration of WNT4 in BJAB cells does not promote apoptosis, but modulates the cell cycle. A)** Percentage of apoptosis was determined by using Annexin-V-Fluos/Propidium Iodide in BJAB-Tet-WNT4 cells after 48-h incubation in the presence or absence of Doxycycline (Doxy). We also included parental BJAB and BJAB-Tet cells as control. A total of 20,000 events were counted. **B)** Parental BJAB, BJAB-Tet, and BJAB-Tet-WNT4 cells were cultured in presence of Doxy for 48 h. Afterward, flow cytometric analysis of the DNA content was conducted to determine cell populations in the different cell-cycle phases. Dotplots and histograms were obtained with Attune Cytometric Software ver 2.1.0, G1 and G2 populations are shown. **C)** Histograms obtained by analyzing cell cycle with FlowJo software. A total of 20,000 events in gated singlets were counted.

A question that emerged from the previous set of experiments was whether the decreased viability induced by WNT4 expression in BJAB cells was due to an inhibition in cell proliferation or an induction of apoptosis. To determine this, we measured the apoptotic rate of parental BJAB, BJAB-Tet, and BJAB-Tet-WNT4 cells grown in the presence of Doxy to induce WNT4 expression. Figure 5A shows that Doxy alone exerts no effect on apoptosis in parental BJAB or BJAB-Tet cells, even after 48 h of incubation (Figure 5A). Interestingly, the WNT4 expression triggered by Doxy in BJAB-Tet-WNT4 cells induces a slight, but not significant, increase in the apoptosis rate (only 2%). Because we did not observe a considerable impact on apoptosis, our next interest was to elucidate whether WNT4-induced expression modulates the cell cycle. To address this point, parental BJAB, BJAB-Tet, and BJAB-Tet-WNT4 cells were cultured in the presence of Doxy for 48 h to maintain the same conditions for WNT4 expression, then, we analyzed total DNA content using flow cytometry to determine the size of cell populations in the different cell-cycle phases. It is worth noting that prior to seeding the cells for this experiment, cells were cultured for 24 h with low levels (2%) of Fetal bovine serum (FBS) in an attempt to synchronize their growth before being seeded as usual with 10% FBS. As shown in Figure 5B, 61.98% of parental BJAB cells accumulated in G1 phase after 48 h Doxy treatment, compared with 49.49% of BJAB-Tet cells and 93.57% of BJAB-Tet-WNT4 cells. The data were analyzed using the cell cycle tool from the Flowjo v7.6.5 software, which showed a more pronounced effect of G1 arrest after Doxy treatment (Figure 5C). These observations led us to conclude that the inhibition of cell proliferation mediated by WNT4 expression is due to cells arresting in the G1 phase of the cell cycle.

### Inhibition of cell proliferation by WNT4 does not activate the canonical pathway

**Figure 6:**
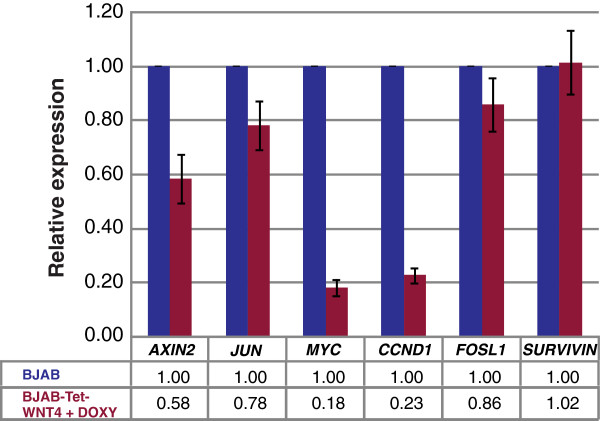
**Inhibition of cell growth through WNT4 did not involve the canonical pathway.** Relative expression levels of *AXIN2*, *MYC*, *JUN*, *CCND1*, *FOSL1*, and *SURVIVIN* were determined by qRT-PCR in parental BJAB and BJAB-Tet-WNT4 cells. Quantification was calculated by normalizing with BJAB (set as 1) and using *ACTB*, *RPS18*, and *RPL32* as reference genes. The graph shows the means obtained with reference genes ± Standard deviations (SD).

To determine whether the growth inhibition in BJAB cells observed after WNT4 restoration was mediated by activation of the canonical β-catenin pathway, we analyzed the expression of several reported target genes for this pathway by qRT-PCR. We determined the mRNA expression of *AXIN2*, *JUN*, *MYC*, *CCND1*, *FOSL1*, and *SURVIVIN* in parental BJAB and in BJAB-Tet-WNT4. As can be observed in Figure 6, none of the previously mentioned genes were activated by restoring WNT4; instead, nearly all of the genes, with the exception of *SURVIVIN*, demonstrated decreased expression in WNT4-expressing BJAB cells. These results indicate that the effect of WNT4 on BJAB cell growth may not mediated by the stabilization of β-catenin and, consequently, activation of the canonical pathway.

### FZD6, partner of WNT4, is expressed in lymphoid but not myeloid cell lines

FZD6 is the sole Frizzled (FZD) receptor that has been linked to WNT4 in hematopoietic cells [15]; therefore, we thought it important to establish whether the action of WNT4 in our cell model was due to binding to FZD6. First, we measured the mRNA levels of this receptor by qRT-PCR, and then performed expression analyses (∆CP). We found that FZD6 is most clearly detected in BJAB, Jurkat, and CEM cells. Interestingly, myeloid cell lines K562 and HL60 express the lowest levels of FZD6 mRNA (Additional file 1: Figure S1A). To confirm these observations, we additionally detected the FZD6 receptor at the protein level by flow cytometry. As illustrated in Additional file 1: Figure S1B, high expression of FZD6 was observed in lymphoid Jurkat, CEM, and BJAB cell lines. On the other hand, myeloid cell line K562 exhibited very low levels of this receptor, and HL60 levels were undetectable. These results suggest that inhibition of cell proliferation mediated by WNT4 may not be dependent on the binding of this ligand to FZD6, especially not in cell lines of myeloid origin.

## Discussion

WNT-mediated signal transduction pathways have long been recognized for their roles in regulating embryonic development; this pathway additionally plays a prominent role in stem cell biology, including self-renewal, pluripotency, and differentiation [7,24]. However, more recently it has also been linked to cancer and human disease processes [2,25]. Specifically, in leukemia there is experimental evidence demonstrating the influence of WNT pathway components on the oncogenic growth of disease from both myeloid and lymphoid origin [7-10,12], but there is a very limited number of publications on WNT4 expression and its role in the biology of these cells. Therefore, we were interested in probing deeper into the role that WNT4 plays in malignant hematopoietic cells.

Our results show that WNT4 is expressed in normal cells, predominantly in B-cells, but that its expression is strongly reduced in leukemia-derived cell lines and in BM cells from patients with leukemia. In accordance with this observation, Lu et al. reported that WNT4 levels are decreased in patients with Chronic lymphocytic leukemia (CLL), compared with healthy control B-cells. It has been reported that WNT4 is poorly expressed in endometrial and squamous cell carcinomas [18,26]; additionally, it has been observed that WNT4 is downregulated in human anaplastic thyroid carcinomas [27]. In contrast, Memarian A, *et al*., using semi-quantitative PCR, found higher expression of WNT4 in patients with CLL when compared with that of normal subjects [22]; however, the authors did not find this difference in patients with Acute lymphoblastic leukemia (ALL) [23]. The different WNT4 expression pattern observed between normal and the malignant cells suggests that this ligand could be essential for normal cell development. Regarding WNT4 protein expression level, it is important to note that we observed significant WNT4 protein expression in PBMCs, but relatively lower than the amount observed at the mRNA level. We hypothesized that this may be due to the fact that B lymphocytes are the major WNT4-expressing cells, which comprise only 5 – 15% of the lymphocytes in the PBMC population. Additionally, it could be that this protein is actively secreted, for we did not use any secretion inhibitor in our experiments.

To our knowledge, our group is the first to report that WNT4 inhibits the cell growth of leukemia-derived cell lines. We were able to demonstrate this effect by restoring WNT4 using an inducible lentiviral overexpression system and the addition of a commercially available recombinant human WNT4 protein. Some reports support the anti-proliferative action of non-canonical WNT ligands in cancer; for example, it has been demonstrated that restoration of WNT7a expression reverses cellular transformation in non-small cell lung cancer (NSCLC) by mediating growth inhibition and promoting cell differentiation. Inhibition of cell proliferation due to the restoration of WNT7a has also been observed in PC12 cells and leukemia-derived cell lines [28-32]. In contrast, WNT4 has been reported to enhance murine hematopoietic progenitor cell expansion [15] and, interestingly, overexpression of WNT4 has been associated with differentiation in human primary endometrial stromal cells (HESCs) [33]. It has been also reported that WNT4 interferes with Ras-induced actin cytoskeleton reorganization; it is known that aberrant motility and invasive ability are relevant hallmarks of malignant tumor cells [27].

Although we determined WNT4 inhibited cell proliferation in our cell culture model of inducible WNT4 expression, no effect on apoptosis was observed. It appears that WNT4 expression strongly modulates the cell-cycle phases, because an arrest in G1 and S phases was observed after the restoration of WNT4 expression. Contrary to our findings, Heinonen *et al*. also found that WNT4 highly induces the anti-apoptotic protein Bcl-_XL_, but these authors did not find differences in cell-cycle phases [15]. The discrepancy in these findings could be caused by the use of different research models: while our research has been conducted in human leukemia-derived cells, Heinonen *et al.* employed a murine model.

By measuring diverse *WNT* target genes, we were able to determine that inhibition of cell growth through WNT4 does not involve the canonical pathway. There is increasing evidence that WNT4 signals through a non-canonical pathway in many cell types, such as β-cells [34], human anaplastic thyroid carcinomas [27], murine hematopoietic progenitor cells [15], and human pituitary adenomas [35]. However, in Madin-Darby canine kidney (MDCK) epithelial cells [17] and in endometrial stromal cells [33], it has been observed that WNT4 operates via both the non-canonical and the canonical pathways. Another report demonstrates that WNT4 inhibits β-catenin/TCF signaling by redirecting beta-catenin to the cell membrane [36].

Because the effects of *WNT* signaling are strongly dependent on the nature of the Frizzled receptors present [37], it was of great interest to us to attempt to elucidate the receptor that was involved in the inhibition of cell proliferation. To date in the scientific literature, it has been reported that WNT4 binds to the FZD6 receptor to induce canonical signaling [15,17]; more recently, it was reported to also bind to PTK7/Otk to induce non-canonical signaling [38]. We report here that WNT4 cell-growth inhibition in leukemia-derived cells appears to be FZD6-independent, at least in myeloid cell lines (K562 and HL60), because this receptor is not expressed in these cells at the protein level. A clear effect on the inhibition of proliferation was observed in all cases. In contrast to our observations, FZD6 expression has been reported to be present in K562 and HL60 by other authors [21,39]; however, in both reports, FZD6 expression was analyzed only at the mRNA level (Northern blot and PCR). This suggests that other receptors/co-receptors must be involved in WNT4 signaling and that the molecules involved are dependent on the cell type.

We suggest that the maintenance of appropriate WNT4 expression may also be critical for prevention against tumor initiation. Interestingly, WNT4 overexpression in stromal cells (OP9-DL1–W4) was sufficient to allow LN c-Kit^lo^Sca-1^+^ cells to generate mature T-cells [40]. It could be that the inhibition of WNT4-mediated proliferation in leukemia-derived cells is due to the regulation of genes that participate in controlling proliferation and differentiation.

## Conclusions

We showed that WNT4 expression is strongly reduced in leukemia-derived cell lines and in blasts from patients with leukemia compared with the expression in mature blood cells from healthy individuals. Interestingly, restoration of WNT4 expression inhibits cell growth in a non-canonical manner and appears to be FZD6-independent. Our results suggest that WNT4 could act as a tumor suppressor for leukemia by antagonizing WNT/β-catenin signaling.

## Methods

### Ethics statement

This protocol was approved by Ethical and Research Committee No. 1305 of the Centro de Investigación Biomédica de Occidente (CIBO) – Instituto Mexicano del Seguro Social (IMSS), with registration number: R-2011-1305-6. Authorization for the taking samples from patients with leukemia was obtained from the National Health Research Committee (IMSS), with registration number: R-2012-785-056. Written informed consent from healthy volunteers and patients with leukemia (in compliance with the Helsinki Declaration) was also required prior to blood/bone marrow sample collection.

### Cell line culture

Human leukemia-derived cell lines BJAB (lymphoblasts of acute B-cell leukemia- derived cells), Jurkat, CEM (lymphoblasts of acute T-cell leukemia), K562 (lymphoblasts of erythroleukemia), and HL60 (promyeloblasts of acute promyelocytic leukemia) were employed as study models. Cells were suspended in RPMI medium-1640 supplemented with 10% Fetal bovine serum (FBS), penicillin (100 U/mL), and streptomycin (100 μg/mL) at 37°C in a humidified atmosphere of 5% CO_2_. All of the previously mentioned products were obtained from the GIBCO™ Invitrogen Corporation.

### Isolation of PBMC, bone marrow, and T- and B-cells

Peripheral blood mononuclear cells (PBMCs) obtained from five healthy volunteers (10 mL of peripheral blood) were isolated by density-gradient centrifugation with Ficoll-Paque™ PLUS (GE Healthcare). The PBMCs were resuspended in PBS and stained with an anti-CD3 antibody (sc-1179-FITC, Santa Cruz Biotechnology) to select T-lymphocytes and an anti-CD19 antibody (sc-19650-PE, Santa Cruz Biotechnology) to select B-lymphocytes. After incubation with both antibodies, cells were washed, and cells positive for CD3 or CD19 were sorted on a FACSAria (Becton Dickinson). Bone marrow samples (1–2 ml) from patients with leukemia were also obtained by density-gradient centrifugation with Ficoll. General characteristics of patients with leukemia are included in Additional file 2: Table S1.

### Restoration of WNT4 with recombinant human protein

BJAB, Jurkat, CEM, K562, and HL60 cells were cultured at a density of 0.5 × 10^4^ cells in 96-well microtiter plates in 200 μL of RPMI medium. Recombinant human WNT4 (cat. no. 6076-WN; R&D Systems) was reconstituted in sterile Phosphate-buffered solution (PBS). The recombinant protein was added at a final concentration of 200 ng/mL; incubations at 37°C were performed for 24 and 48 h.

### Cloning WNT4

The WNT4 open reading frame (ORF) (GeneID: 54361; NM_030761.4) was amplified from epithelial cells derived from human thymoma using the Expand High Fidelity PCR System (cat. no. 11 732 650 001; Roche Applied Science) with the following set of primers: forward 5′- GGC ACC ATG AGT CCC CGC TCG -3′, and reverse: 5′- GCA GGG CTA GGC AGG CGG TCA -3′. Afterward, the PCR product was run on a 1% agarose gel and purified with the Vivantis GF-1 Gel DNA Recovery kit (SKU GF-GD-050). The WNT4 ORF was cloned into pGEM®-T Easy vector (cat. no. A1360).

The resulting construct was sequenced with the M13 Forward and Reverse primers (Invitrogen) using the Big-Dye® Terminator Cycle Sequencing Kit (Applied Biosystems). WNT4 ORF was isolated from the pGEM®-T Easy vector using EcoRI restriction and subcloned into the EcoRI site of the lentiviral expression vector pLVX-Tight-Puro (cat. no. 632162; Clontech Laboratories, USA). Prior to cloning, this vector was previously unphosphorylated.

### Lentivirus production and infection

To produce infectious viral particles, Lenti-X 293 T-cells were transfected employing the Lentiphos HT™ Packaging System (cat. no. 632151; Clontech Laboratories) with lentiviral vectors pLVX-Tet-On Advanced and pLVX-Tight-Puro-WNT4, which were used as described by the manufacturer (Clontech Laboratories). After 48 h, the supernatants were collected and checked with Lenti-X GoStix (Clontech Laboratories) to determine whether sufficient viral particles were produced prior to transducing target cells. The supernatants were filtered through a 0.45-μm PES filter to eliminate detached cells, aliquoted, and subsequently stored at -80°C until use. BJAB cells were first transduced with the pLVX-Tet-On (regulator vector) and selected with G418 (cat. no. 631307; Clontech Laboratories), 500 μg/mL. The cells were next transduced with pLVX-Tight-Puro-WNT4 and selected with Puromycin for 2 weeks (1 μg/mL). After selection, cells were grown in the absence or presence of Doxy (750 ng/mL) to overexpress WNT4.

### Primer design

Primer design for the WNT4 ligand was performed using Oligo-Primer Analysis ver. 6.51 software (Molecular Biology Insights, Inc., USA) using sequences obtained from the GenBank nucleotide database on the NCBI website. Specific primers and some of their features are listed in Table 1. Primers were synthesized by the Invitrogen Corporation.

**Table 1:** Oligonucleotides used for the qRT-PCR analysis

	**Gene name**	**Sequence accession number**	**Primer sequence**	**Primer location (Exon)**	**Prod. length**	**T°a**
***WNT4 ORF***	Wingless-type MMTV integration site family, member 4	NM_030761	**F** GGCACCATGAGTCCCCGCTCG	99–119 (1)	1055	60
			**R** GCAGGGCTAGGCAGGCGGTCA	1158–1178 (5)		
***WNT4***	Wingless-type MMTV integration site family, member 4	NM_030761	**F** GGAACTGCTCCACACTCGACTC	364–385 (3)	259	60
			**R** CGCACATCCACAAACGACTGT	602–622 (4)		
***MYC***	V-myc myelocytomatosis viral oncogene homolog (avian)	NM_002467.4	**F** CCAGCGCCTTCTCTCCGTC	1208–1226 (2)	302	60
			**R** GGGAGGCGCTGCGTAGTTGT	1490–1509 (3)		
***JUN***	Jun proto-oncogene	NM_002228.3	**F** TGGAAAGTACTCCCCTAACCT	2786–2806 (1)	250	60
			**R** CTGAAACATCGCACTATCCTT	3015–3035 (1)		
***FOSL1/FRA1***	FOS-like antigen 1	NM_005438.3	**F** AGGAACCGGAGGAAGGAACTG	554–574 (3)	199	60
			**R** TGCCACTGGTACTGCCTGTGT	732–752 (4)		
***AXIN2***	Axin 2	NM_004655.3	**F** AAAAAGGGAAATTATAGGTATTAC	2678–2701 (10/11)	277	54
			**R** CGATTCTTCCTTAGACTTTG	2935–2954 (11)		
***CCND1***	cyclin D1	NM_053056.2	**F** CCCCAACAACTTCCTGTCCTAC	866–887 (4)	236	60
			**R** GCCCTCAGATGTCCACGTC	1083–1101 (5)		
***SURVIVIN/BIRC5***	Homo sapiens baculoviral IAP repeat containing 5	NM_001012271.1	**F** TGAGCTGCAGGTTCCTTATCTG	1057–1078 (5)	234	60
			**R** GAATGGCTTTGTGCTTAGTTTT	1269–1290 (5)		
***RPL32***	Ribosomal protein L32	NM_000994.3	**F** GACTTGACAACAGGGTTCGTAG	213–234 (3)	320	60
			**R** ATTTAAACAGAAAACGTGCACA	511–532 (4)		
***RPS18***	Ribosomal protein S18	NM_022551.2	**F** CGATGGGCGGCGGAAAA	105–121 (2)	283	58
			**R** CAGTCGCTCCAGGTCTTCACGG	366–387 (5)		
***ACTB***	Beta Actin	NM_001101.3	**F** TCCGCAAAGACCTGTACG	950–967 (5)	298	60
			**R** AAGAAAGGGTGTAACGCAACTA	1226–1247 (6)		

### qRT-PCR assays

Total RNA was isolated from 5 × 10^6^ BJAB cells transfected with the WNT4 DNA construct at 0, 4, 8, 16, and 24 h after treatment with Doxy. Isolation was performed using the PureLink™ Micro-to-Midi Total RNA Purification System (cat. no. 12183–018; Invitrogen) as suggested by the manufacturer.

Total RNA was reverse-transcribed to cDNA using the SuperScript™ III First-Strand Synthesis System primed with oligo(dT) (cat. no. 18080051; Invitrogen). cDNA synthesis was performed from 5 μg of total RNA. The protocol was carried out as suggested by the manufacturers.

Gene expression levels were analyzed by qRT-PCR. Assays were performed with 2.0 LightCycler technology using the LightCycler FastStar DNA Master PLUS SYBR Green I kit (cat. no. 03515885001; Roche Applied Science) as recommended by the manufacturers. Analysis of gene expression was performed with LightCycler ver. 4.1 software (LCS). *RPS18* (d/ing: 18 s Ribosomal Protein), *RPL32* (d/ing: ribosomal protein L32), and *ACTB* (d/ing: Actin beta) were used as endogenous controls. Relative quantification of target genes was determined using the ΔΔCP method.

FZD6 expression analysis was carried out by ∆CP (FZD6 CP - reference gene CP). It is very important to point out that ∆CP is inversely proportional to the expression of the target gene.

Analysis was performed using the values obtained from two independent RNA extractions done in duplicate.

### Western blot assays

Cells were lysed with RIPA buffer by sonication (15 pulses, 90% amp). Total extracts were incubated for 30 min at 4°C and subsequently obtained by centrifugation (14,000 rpm for 5 min at 4°C). Protein concentrations were determined using the Bio-Rad DC Protein kit (cat. no. 500–0114, Protein DC-BioRad; Bio-Rad Laboratories), and 50 μg of the extracts was electrophoresed in 12% SDS-PAGE. Proteins were then transferred onto a PVDF membrane (Millipore) and incubated with 1% Western blocking reagent (cat. no. 11921681001; Roche Applied Science) to block nonspecific binding. Primary antibody (anti-WNT4: cat. no. MAB4751, R&D Systems; Actin: cat. no. sc-1616, Santa Cruz Biotechnology; α tubulin: cat. no. sc-8035, Santa Cruz Biotechnology and anti-beta 2 microgloblin: cat. no. ab15976, Abcam) was incubated overnight at 4°C, and specific secondary antibody was incubated with the membrane for 1 h at room temperature, followed by chemiluminescent detection using Immobilon Western substrate (Millipore Corporation) with the ChemiDoc XRS (Bio-Rad Laboratories).

### Measurement of cell viability

Cell viability was determined using WST-1 (cat. no. 11 644 807 001; Roche Applied Science). Absorbance of treated and untreated cells was measured at 450 nm on a microtiter plate reader (Synergy™ HT Multi-Mode Microplate Reader; Biotek, Winooski, VT, USA). The value of untreated cells was used as 100% cell survival.

### Apoptosis detection

Cell death was measured by flow cytometry using propidium iodide (cat. no. P4864; Sigma-Aldrich) and Annexin-V-FLUOS (cat. no. 1828681; Roche Applied Science) as recommended by these manufacturers. Cells were seeded at a density of 2.5 × 10^5^ cells per flask in 10 mL RPMI medium with or without Doxy (750 ng/mL). After a 72-h incubation, cells were washed with PBS and incubated with annexin and propidium iodide for 15 min; 20,000 events from each sample were analyzed in an Attune Acoustic Focusing Cytometer (AB Applied Biosystems).

### Cell cycle analysis

For cell cycle analysis, we used 2 different kits: the BD Cycletest™ Plus DNA Reagent kit (cat. no. 340242; Becton, Dickinson and Company), and the Cell Cycle Assay kit (Fluorometric-Green, cat. no. ab112116, Abcam). Both were used following the manufacturer’s instructions. DNA QC particles (cat. no. 349523) were used for verification of instrument performance and quality control of BD FACS™-brand flow cytometer employed in the DNA analysis. For each sample, at least 20,000 events were acquired in the singlet’s region and data were analyzed using the cell cycle tool from the Flowjo v7.6.5 software package (Tree Star Inc., OR, USA).

### Flow cytometry analysis

A total of 1 × 10^6^ cells were suspended in 100 μL of Phosphate-buffered saline (PBS), and anti-human Frizzled-6 antibody (cat. no. AF3149; R&D Systems) was added. After 30 min at room temperature, the cells were washed once and resuspended in PBS. Then, phycoerythrin-conjugated donkey anti-goat IgG (cat.no. F0107; R&D Systems) was added. We incubated the cells for an additional 30 min. Finally, the cells were washed twice and were read in the cytometer.

### Statistical methods

The data obtained are shown as mean ± Standard deviation (SD). Post-hoc tests (Tukey HSD, Bonferroni, and Dunnett 3 T) were utilized for multiple comparisons between groups and one-way analysis of variance (ANOVA) was employed to compare means between more than two different groups. Comparison between two groups was performed using a two-tailed T-test. Only *p* values of <0.05 were considered significant. Statistical analysis was performed with Predictive Analytic SoftWare (PASW) (Statistics ver. 18.0).

## Competing interests

The authors declare that they have no competing interests.

## Authors’ contributions

BGC was closely involved in most of the experiments shown, and contributed to the writing of the manuscript. MAZ performed flow cytometry, qRT-PCR experiments and western blots. POL and SRG performed flow cytometry sorting; ARM recruited patients with leukemia and measured WNT4 expression in their cells. GHF and ABC contributed to the planning of the project and provided scientific suggestions. LFJS and AAL conceived of and designed the theoretical framework of the study, provided scientific guidance throughout the project, and contributed to the writing of the manuscript. All authors approved this final version.

## Pre-publication history

The pre-publication history for this paper can be accessed here:

http://www.biomedcentral.com/1471-2407/13/557/prepub

## Supplementary Material

Additional file 1: Figure S1 FZD6, partner of WNT4, is expressed in lymphoid but not myeloid cell lines. A) FZD6 expression levels (∆CP) measured by qRT-PCR in normal PBMCs and leukemia-derived cell lines (BJAB, Jurkat, CEM, K562, and HL60). ∆CP values were calculated utilizing ribosomal Protein L32 (*RPL32*) and ribosomal Protein S18 (*RPS18*) as reference genes. The graphs depict the means and subsequent standard deviations (SD) obtained with all reference genes. B) Representative histograms of flow cytometry data for FZD6. Numbers at the upper part of each curve represent the geometric median for each condition: control unlabeled cells (red curve); secondary antibody (black curve), and FZD6 antibody (green curve). Click here for file

Additional file 2: Table S1 General Characteristic of patients with leukemia. Click here for file
