# Supplementary figures and images for: Restoration of WNT4 inhibits cell growth in leukemia-derived cell lines
Source: BMC Cancer. 2013 Nov 25;13:557. doi: 10.1186/1471-2407-13-557 (PMC4222640; doi:10.1186/1471-2407-13-557)

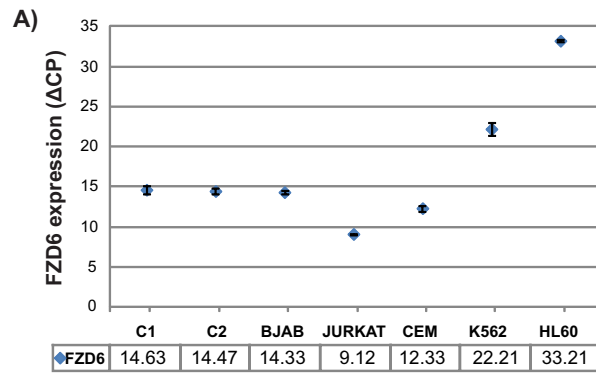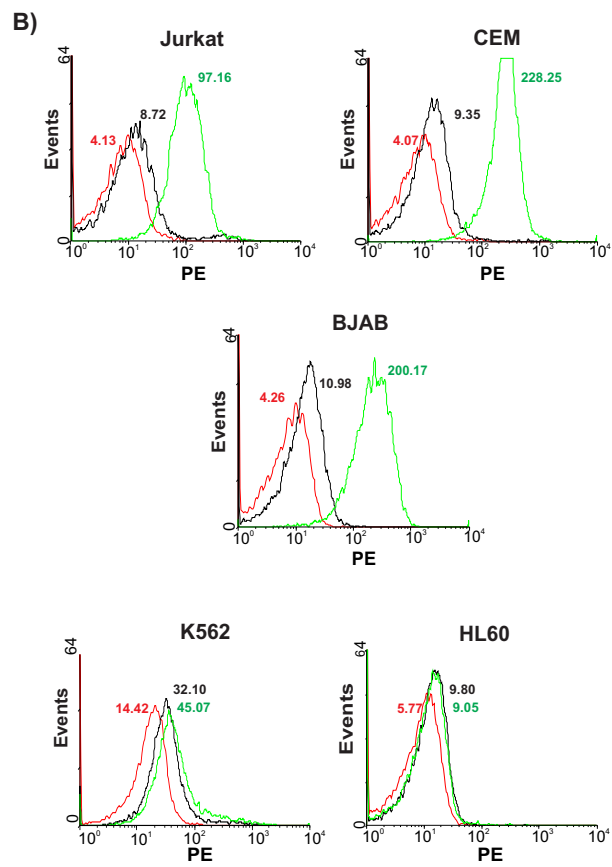

Supplement: Additional file 1: Figure S1 — FZD6, partner of WNT4, is expressed in lymphoid but not myeloid cell lines. A) FZD6 expression levels (∆CP) measured by qRT-PCR in normal PBMCs and leukemia-derived cell lines (BJAB, Jurkat, CEM, K562, and HL60). ∆CP values were calculated utilizing ribosomal Protein L32 (RPL32) and ribosomal Protein S18 (RPS18) as reference genes. The graphs depict the means and subsequent standard deviations (SD) obtained with all reference genes. B) Representative histograms of flow cytometry data for FZD6. Numbers at the upper part of each curve represent the geometric median for each condition: control unlabeled cells (red curve); secondary antibody (black curve), and FZD6 antibody (green curve). [file 1471-2407-13-557-S1.pdf]
