# Supplementary material for: Restoration of WNT4 inhibits cell growth in leukemia-derived cell lines
Source: BMC Cancer. 2013 Nov 25;13:557. doi: 10.1186/1471-2407-13-557 (PMC4222640; doi:10.1186/1471-2407-13-557)
Supplement: Supplementary file 2 [file 1471-2407-13-557-S2.doc]

Supplemental Table 1. General characteristics of patients with leukemia

Patient ID	Gender	Age (years)	Immuno-phenotype	BM blast infiltration (%)
5	F	49	LLA-B	67.9%
M7	M	27	LMA M0-M1	89.3%
M8	M	19	LMA M5	92.4%
M10	F	41	LMA M0-M1	83.7%
M11	F	38	LMA M0	61.7%
M12	M	77	LLA-T	96.0%
M14	M	56	LMA M0	83.7%
M15	M	17	LLA-B	94.9%
M18	M	40	LMA M5	77.5%
M27	M	23	LMA M5	98.0%
M28	M	16	LLA-B	80.0%
